# Mass mortality associated with koi herpesvirus in common carp in Iraq

**DOI:** 10.1016/j.heliyon.2020.e04827

**Published:** 2020-08-31

**Authors:** Mustafa Ababneh, Wael Hananeh, Mohammad Alzghoul

**Affiliations:** a Department of Basic Medical Veterinary Sciences, Jordan University of Science & Technology, P.O. Box 3030, Irbid, 22110, Jordan; b Department of Pathology and Public Health, Jordan University of Science & Technology, P.O. Box 3030, Irbid, 22110, Jordan

**Keywords:** Microbiology, Virology, CyHV-3, Mass mortalities, Common carp, Iraq

## Abstract

Koi herpesvirus disease is a serious disease affecting both wild and common carp species in different continents throughout the world. Based on pathological and molecular findings, we document the presence of koi herpesvirus disease in Iraq as a cause of mass mortality among the common carp of the Tigris river. On a macroscopic level, the fish exhibited variably sized skin ulcerations throughout the entire trunk. The gills showed variable degrees of discoloration with an increased amount of slimy mucus. Microscopically, degeneration and necrosis with infiltration of a heterogenous population of inflammatory cells characterized different organs, primarily the skin and gills, with occasional intranuclear inclusion bodies that are consistent with koi herpesvirus disease. A semi-nested PCR assay coupled with sequencing confirmed the pathological diagnosis. Genotyping and sequence analysis of the TK gene, ORF 136 and markers I and II identified the isolated CyHV-3 as variant A1 of the Asian genotype TUSMT1 (J strain) displaying the I^++^II^+^ allele.

## 1 Introduction

Koi herpesvirus disease (KHVD), a serious disease affecting both wild and common carp species, is caused by the cyprinid herpesvirus 3 (CyHV-3) [1]. KHVD can occur on its own or as part of a coinfection alongside other viral agents such as carp edema virus [2]. Listed as a notifiable disease according to the International Office of Epizootics, KHVD first appeared in Germany in 1997 and soon spread to many countries in Europe, Asia, North America and Africa, causing huge losses in common carp and koi culture production [1]. CyHV-3 is highly contagious and extremely virulent, having a mortality rate of up to 80%–100% [1]. In fact, carp infected with CyHV-3 and kept at 23–28 °C die between 5 and 22 days post infection (dpi), with peak mortality occurring between 8 and 12 dpi [1]. Furthermore, the virus can persist for some period of time in a latent or carrier state without obvious clinical signs [1].

Belonging to the genus *Cyprinivirus*, CyHV-3 is a double-stranded DNA virus with a linear 295 kbp genome [3]. Different strains of CyHV-3 have been identified: a Japanese or Asian strain (strain J), European strains that include the Israel (strain I) and United States (strain U) strains [3]. Strains I and U are more closely related to each other than to other strains, although a unique Chinese CyHV-3 isolate (GZ11) was found to be closer to the European genotype [4]. The Asian strain is highly homogenous, with only two variants (A1 and A2), while the two European strains include seven variants (E1–E7) [5]. A duplex PCR assay has been developed to identify the I, J, and U strains based on differences in three variable domains within two genetic markers, namely markers I and II [6]. This duplex PCR technique classifies the different CyHV-3 strains based on the presence (+) or absence (−) of each of the three variable domains [6]. U/I strains are designated I^−−^II^−^ as all three domains are absent in markers I and II, while the J strain is designated I^++^II^+^ as all three domains are present [6]. Recently, a new genotype (I^−+^II^−^), which has one of the two marker I domains, was reported in the Netherlands, and, in Malaysia, another novel genotype with a deletion of 13 nucleotides in marker II (I^++^II^+Δ^) was identified [7, 8].

According to the Food and Agriculture Organization of the United Nations, freshwater fish production reached 46.6 million tons in 2016, with cyprinids constituting almost 65% of this annual total [9]. Carp is considered to be an important source of food in many parts of Asia, as China and India account for 72.8% and 13.8% of global carp production, respectively [9]. Freshwater fish production is a flourishing industry in Iraq, where, due to a limited coastline bordering the Gulf, the Tigris and Euphrates rivers serve as the main areas for farming freshwater fish [9]. Freshwater fish farms in Iraq can be found as a part of either the public or private sectors, and they are mainly located in the central and southern parts of Iraq [10]. Freshwater fish production is limited to the culture of common carp (*Cyprinus carpio carpio*), but there is also a limited culture of grass carp (*Ctenopharyngodon idella*) and silver carp (*Hypophthalmichthys molitrix*) [10]. In 2015, the total freshwater carp production of Iraq was estimated to be approximately 30,000 tons [10]. In late October 2018, a major KHVD outbreak hit carp cultures in Iraq's central Euphrates region [11]. Soon thereafter, in early November 2018, our laboratory received dead fish for histopathological and molecular examination to investigate the possible causes of this outbreak. The results of our work, which are detailed in this study, indicate the presence of CyHV-3 in those carp samples.

## 2 Materials and methods

Approval from the Jordan University of Science and Technology Animal Care and Use Committee (JUST-ACUC) is not applicable in this instance as all carp samples were submitted to our laboratory for diagnostic investigation after their death.

### 2.1 Clinical findings and history

In late October 2018, mass mortality of common carp occurred in carp farms. Our laboratory received a total of five dead fish from a private farm located on the Tigris River. The private farm is located in the north of Bagdad in the town of Tarmiyah, this private farm has 15 caged ponds, each pond has around 50 smaller cages, with carp of different ages were raised in those cages. The estimated carp population in this farm was about 2 million fish. Four of the dead fish were submerged in 10% formalin but improperly fixed, while the fifth fish was submitted in a frozen state. Samples were delivered to our laboratory two days after collection, and, according to a local veterinarian, the following behavioural changes were observed in the affected fish before their death: lethargy, lack of appetite, itching behaviour on cage walls, and a general presence near the water surface. Moreover, increased mucus was observed on the body surfaces along with red spots on the fins, whitish patches on the skin, discoloration and necrosis of the gills, and sunken eyes. Mortality was 100% in all affected ponds in fish of different ages. The dead fish that were submitted to our laboratory underwent histopathological and molecular examination, which involved the collection of different tissue samples from the skin, gills, liver, kidney, heart, spleen, pancreas, brain and intestine.

### 2.2 Histopathological examinations

Relevant tissue samples were collected from the four carps submerged in 10% formalin, and all samples were processed routinely in an automatic tissue processor and then embedded in paraffin. 4- μm tissue sections were cut and stained with haematoxylin and eosin (H&E) stain (H&E), and the stained tissues were evaluated by a certified veterinary pathologist.

### 2.3 Molecular examination

Different assays were employed to diagnose and genotype the causative agent behind this outbreak. For diagnosis, a one-tube, semi-nested PCR (snPCR) was performed to detect CyHV-3 in certain fish tissues, namely the skin, gills, liver, kidney, heart, spleen, brain and intestines. The PCR products of this assay were then sequenced.

For genotyping, four PCR protocols coupled with sequencing were performed.

Genomic DNA from fish tissues were isolated as follows: fish tissues were cut into small pieces and then homogenized using an Omni Bead Ruptor 24 Bead Mill Homogenizer (Omni International, USA). Genomic DNA was extracted from the above-mentioned tissues using DNeasy Blood & Tissue Kits (Qiagen, Germany) according to the manufacturer's instructions. A diagnostic one-tube snPCR was used to detect the CyHV-3 virus, and this PCR protocol, which targeted the CyHV-3 major glycoprotein gene (ORF 56), was performed according to a previously published study [12]. Positive results in the snPCR showed three bands with sizes of 464, 372, and 182 bp. Similarly, to detect the presence of carp edema virus, a nested PCR protocol was performed to target a partial sequence from the 4a gene according to a previously published study [13].

### 2.4 Sequencing and sequence analysis

The snPCR product band of size 464 bp was excised, gel-purified, and sequenced using a Big Dye 3.1 Terminator Kit (Applied Biosystems, USA) and a SeqStudio Genetic Analyser (Thermo Fisher Scientific, USA). Sequences were edited using the BioEdit software (version 7.2), and one sequence was submitted to GenBank with an accession number of MK636574.

Four different genotyping PCR assays were performed to type CyHV-3 to the right strain. PCR assays targeting the thymidine kinase (TK) gene and ORF 136 were performed using the published primers and protocols [3, 14]. PCR amplification targeting markers I and II was performed as previously outlined by Bigarre et al. [6]. PCR products were sequenced and analysed as mentioned above, and molecular phylogenetic analysis was performed by the maximum likelihood method based on the Tamura 3-parameter model in MEGA6 software.

## 3 Results

### 3.1 Pathological findings

**Figure 1:**
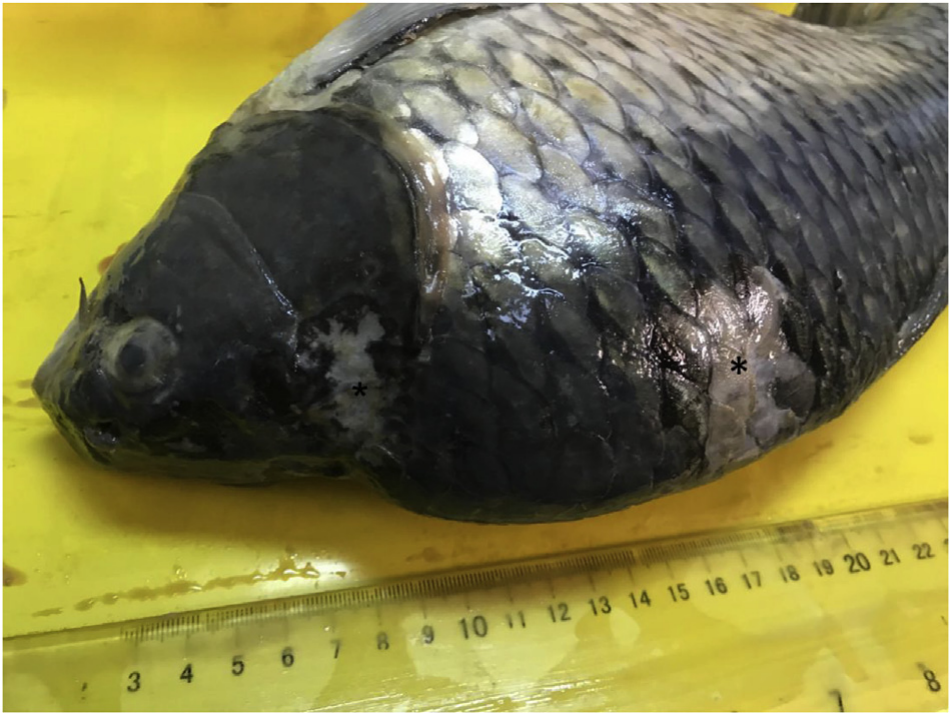
Fish, carp, whole fish. Multiple variably sized and irregular skin ulcers (∗).

**Figure 2:**
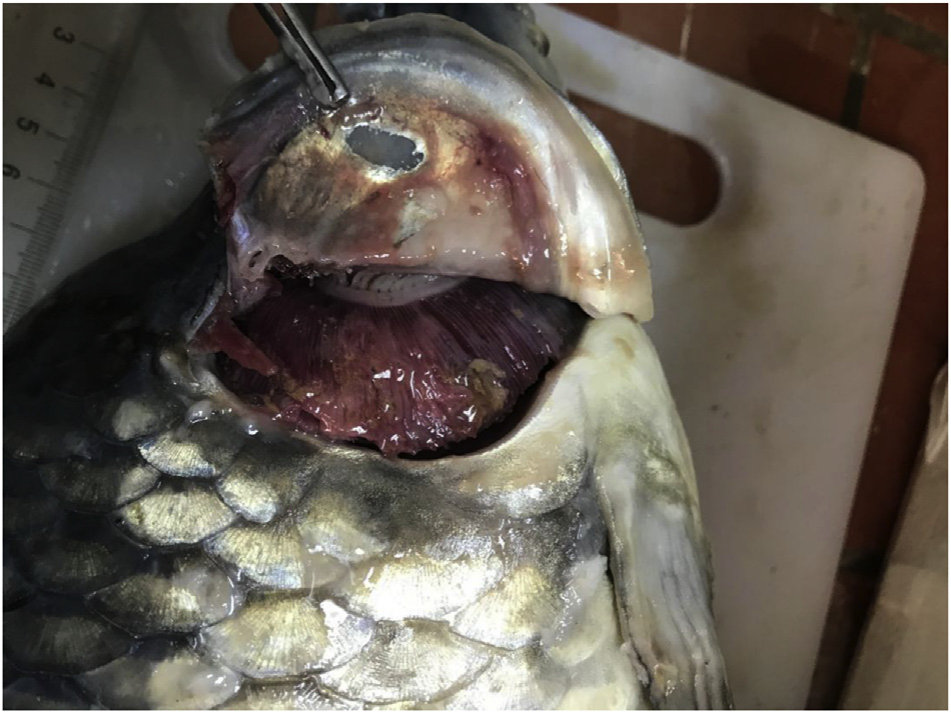
Fish, carp, gill. The gill exhibited variable degrees of discoloration and necrosis.

Upon examination, the gross findings were skin ulcerations of variable sizes throughout the entire trunk (Figure 1). These ulcers were multifocal to coalescing, up to 2 cm in diameter, and with irregular areas of scale and skin loss that exposed underlying subcutaneous tissue and muscles. The largest ulcer was consistently located on the dorsolateral trunk of the affected fish. Upon opening the coelomic cavity, the coelomic organs adhered to each other with fibrinous attachments. The coelomic organs were markedly friable. The gills of the examined fish showed similar gross changes consisting of variable degrees of discoloration and necrosis with an increased amount of slimy mucus (Figure 2). The eyes were markedly sunken. Numerous petechial haemorrhagic areas were spread widely throughout the trunk, primarily around the vent and oral cavity. The fins showed marked erosion with haemorrhages at the base of the fins.

**Figure 3:**
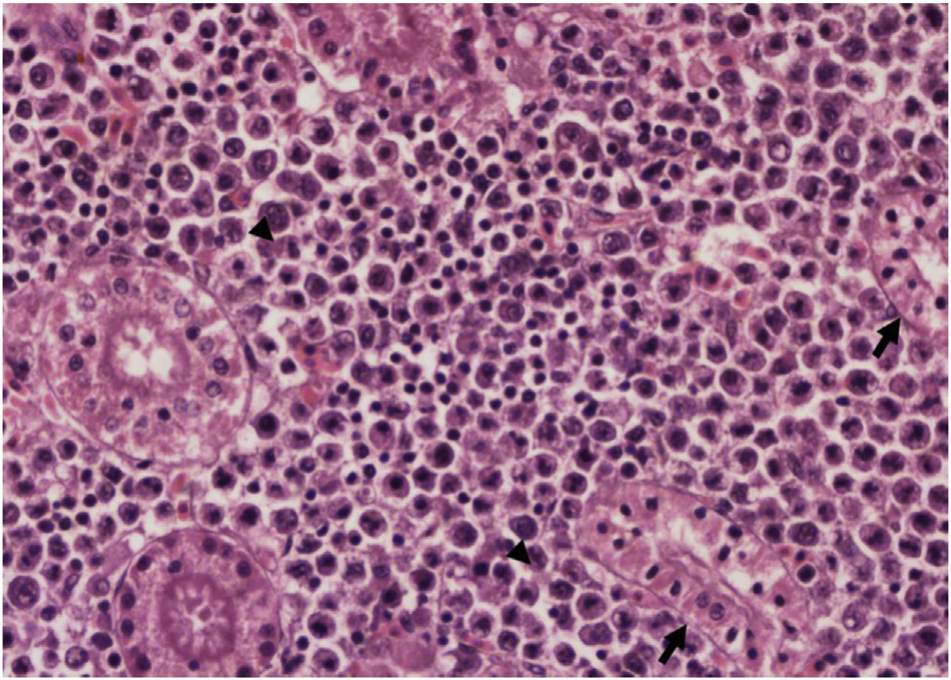
Fish, carp, kidney. Multifocally, the epithelial lining uriniferous tubules exhibited small, round and densely basophilic nuclei (pyknosis) with vacuolated cytoplasm (arrow). Eosinophilic-to-amphophilic intranuclear inclusion bodies were present in the haematopoietic cells (arrow head). H&E stain with 40× magnification.

**Figure 4:**
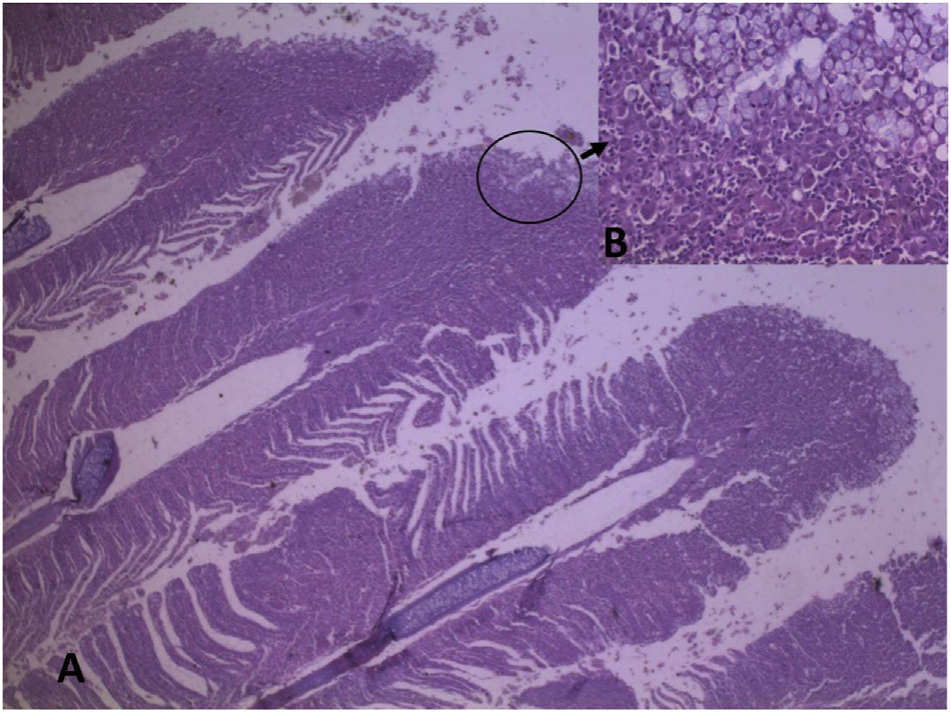
Fish, carp, gill. A. Gill lamellae showed marked fusion of secondary lamellae, and hyperplasia of both epithelial cells covering lamellae and goblet cells with infiltration of mixed inflammatory cells. H&E stain with 4× magnification. Inset B is a higher magnification of A. H&E stain with 40× magnification.

Histopathological examination revealed that the skin was markedly friable. Entire layers of the epidermis were completely lost and infiltrated with a heterogeneous population of inflammatory cells that extended deep into the underlying skeletal muscles. Large numbers of bacterial colonies admixed with fibrinous material, and free erythrocytes were present. The gills exhibited multifocal areas of variable degrees of degeneration and necrosis of the primary and secondary gill lamellae. Other areas of gill lamellae exhibited marked fusions of secondary lamellae and hyperplasia of epithelial cells, as well as goblet cells with severe infiltration of mixed inflammatory cell infiltrates (Figure 3). The interstitium of the primary lamellae was expanded by a heterogeneous population of inflammatory cells, including macrophages, lymphocytes, and eosinophilic granular cells that were mixed with more than one type of protozoal organism. The first protozoa were extracellular, present in moderate numbers, and were 10–20 mm in diameter with a thin cell wall. They had abundant granular-to-vacuolated basophilic cytoplasm with a 5–7 mm round-to oval-single nucleus (amoebic trophozoites). The second protozoa were abundant and scattered throughout the gills. They measured approximately 150 μm in diameter and were round, single-cell protozoal cysts with a 1–2 μm-thick hyaline wall, finely granular-to-vacuolated basophilic cytoplasm containing numerous host erythrocytes, and a 30 × 100 μm, C-shaped, deeply basophilic macronucleus (trophont of *Ichthyophthirius* spp.). These protozoa were also detected in wet mounts of the gills. Occasionally, epithelial lining lamellae exhibited eosinophilic to amphophilic intranuclear inclusion bodies. In the kidneys, multifocally, the epithelial lining uriniferous tubules exhibited small, round and densely basophilic nuclei (pyknosis) with vacuolated cytoplasm. In some areas, total loss of the epithelial lining tubules was present. In the caudal mesonephric kidney sections, frequent eosinophilic-to-amphophilic intranuclear inclusion bodies were present in the haematopoietic cells (Figure 4).

### 3.2 Molecular characterization and phylogenetic analysis

**Figure 5:**
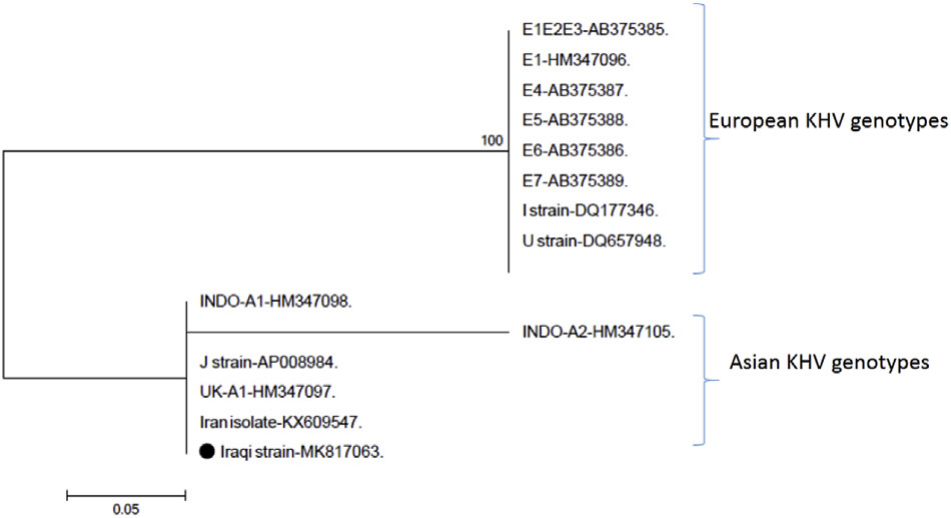
Molecular phylogenetic analysis performed by the maximum likelihood method based on the Tamura 3-parameter model based on the partial nucleotide sequence of thymidine kinase genes of the Iraqi strain and other reference strains of CyHV-3. The scale bar represents a 5% change in nucleotides.

Positive results were detected in the snPCR which showed three bands of 464, 372 and 182 bp lengths. No PCR products were observed in the nested PCR for the carp edema virus in all tested tissue samples. The PCR product band of 464 bp was sequenced and deposited in GenBank under the accession no. MK636574. The sequence was a partial sequence of the koi herpesvirus TK gene. The results of PCR genotyping indicated that the detected CyHV-3 was variant A1 of the Asian genotype TUSMT1 (J strain) and displayed the I^++^II^+^ allele. Molecular phylogenetic analysis performed by the maximum likelihood method based on the Tamura 3-parameter model indicated that the Iraqi CyHV-3 isolate is an Asian genotype (Figure 5). Nucleotide sequence identity between the Iraqi CyHV-3 (GenBank accession no. MK817063) identified in this study and the Iranian isolate of CyHV-3 was 100% in both TK and ORF 136 genes.

## 4 Discussion

This study was carried out to investigate the causative agent behind the mass mortality of carp fish in Iraq. During the preparation of this manuscript, a published article investigated the same problem [15]. Their study was conducted on 12 dead carp samples, 9 of which tested positive in real-time PCR for CyHV-3 and 3 of which tested positive in nested PCR for carp edema virus. In our study, the clinical, pathological, and molecular assays pointed to the sole involvement of CyHV-3, as the nested PCR results for the carp edema virus were negative. The identified CyHV-3 strain was an A1 variant of the Asian genotype TUSMT1 (J strain) and displayed the I^++^II^+^ allele. The same CyHV-3 genotype was implicated in the outbreak that occurred in Iran between 2014–2015, which hit an ornamental koi fish pond in Tehran and caused mass mortality [16]. According to the OIE-Wahis system, there were nine outbreaks from November 2018 to late April 2019 in Iraq [17]. Moreover, between 2018–2019, the OIE-Wahis interface [17] (accessed on October 28th, 2019) reveals that KHVD was reported in Africa, Europe, and North America, an occurrence which might be connected with the global fish trade [1, 18].

The Iraqi KHVD outbreak that started in late October 2018 occurred when the water temperature was optimal for the growth and replication of CyHV-3, i.e. between 18 and 28 °C [19, 20]. The minimum water temperature in Iraq from October to April is in the range between 14.1 and 26 °C, and the maximum water temperature is in the range between 19 and 31.2 °C for the same period. In this outbreak, the clinical manifestation of this disease was similar to that reported in previously published studies. In the Iraqi farms, carp of all ages were affected, a finding which corroborates reports from different natural and experimental studies [21, 22, 23]. The affected carp were lethargic, lost their appetite, and were found near the water surface. Increased mucus was observed on the body surfaces along with red spots on the fin, necrotic areas on the skin, discoloration and necrosis of the gill, and sunken eyes. These clinical signs are typical of KHVD as reported in different studies [16, 24]. Furthermore, the pathological findings in this study were similar to those previously reported for KHVD [16, 24].

The reason behind the mortality of the affected carp is the loss of the osmoregulation function of the gill coupled with massive kidney dysfunction during CyHV-3 infection [25]. The major virulence determinants of CyHV-3 are associated with multiple regions and ORFs. In fact, the ORF 57, ORF 27, ORF 52 and ORF 153 products have been found to be linked to the virulence of CyHV-3 [26,27]. The point-of-entry for the virus is the skin [23], and the changes associated with CyHV-3 infection include dysregulation of genes important to skin barrier function. CyHV-3 has been found to be associated with down-regulation of the mRNA expression of different genes that constitute the mucosal barrier (mucin 5B, beta defensin 1 and 2, and claudin 2, 3c, 11, 23 and 30) [28,29]. Hypersecretion of mucus is noticed in the initial stages of infection, but, in later stages, this secretion is halted.

The high mortality rate of 100% could potentially be explained by the fact that the affected carp farms in Iraq are naïve to the disease, as this is their first exposure to CyHV-3. Moreover, the pathogenesis of the disease could have been complicated by the secondary parasitic infections that were diagnosed in the diseased carp alongside CyHV-3. It has been reported that secondary infections in CyHV-3-diseased carp are common and accentuate the clinical signs of the disease as well as increasing the mortality [30, 31]. While it has been suggested that such secondary infections have no role in the necrotic process of KHVD, they have been observed after the initiation of the necrotic process in the gills [32, 33].

The carp immune response is in a continuous interaction with CyHV-3 infection. In the first 24 h after infection, an increase in the secretion of IFN type 1 (IFN-a1, IFN-a2 and IFN-a1S) is noticed in the skin and spleen of infected carp [29, 34]. While IL-1 beta and inducible nitric oxide synthase (iNOS) appeared at 72 h post-infection and peaked at 120 h post-infection in the skin, lysozyme C (LysC) appeared in the skin 120 h post-infection. Resistance to CyHV-3 infection is also associated with the type of cytokines produced during the infection. A single nucleotide polymorphism (SNP) in IL-10a along with elevated expression of IL-12 p35, IFN ɑβ and TLR 9 contributes to this resistance [35]. This was not the case, however, in a study in which the immune responses in different carp strains with different levels of resistance to KHVD were evaluated: it was found that the resistant Ropsha (Rop) and Amur wild (AS) strains had a higher survival rate than Prerov scale (PS) and koi strains, but the levels of IFN type 1 did not correlate with this resistance phenotype [36].

The exact source and method of introduction of CyHV-3 to carp farms in Iraq are still unknown, but different scenarios can be proposed based on previous studies [16, 37]. The movement of live fish between fish farms and the importation of carp are the most likely routes [37]. Another scenario is that wild non-cyprinid fish can serve as a potential risk factor for CyHV-3 infection [38, 39].

This report confirms the detection of KHVD in Iraq, especially among its freshwater farms. The histopathological and molecular examinations indicated the presence of CyHV-3 in the affected fish, and genotyping and sequence analysis confirmed that the detected virus is a variant A1 of the Asian genotype.

## Declarations

### Author contribution statement

Mustafa Ababneh and Wael Hananeh: Conceived and designed the experiments; Performed the experiments; Analyzed and interpreted the data; Contributed reagents, materials, analysis tools or data; Wrote the paper.

Mohammad Alzghoul: Contributed reagents, materials, analysis tools or data; Wrote the paper.

### Funding statement

This work was supported by 10.13039/501100004035Jordan University of Science and Technology (89/2019).

### Competing interest statement

The authors declare no conflict of interest.

### Additional information

No additional information is available for this paper.
